# Experimental acute muscle pain and itch induce similar inhibitory effects on corticospinal excitability

**DOI:** 10.1097/PR9.0000000000001341

**Published:** 2025-10-14

**Authors:** Bolette Harritsø Winther, Lars Arendt-Nielsen, Johanne Bagger Graugaard, Hannah Grønlund Jespersen, Jesper Elberling, Enrico De Martino, Silvia Lo Vecchio

**Affiliations:** a Center for Neuroplasticity and Pain, Department of Health Science and Technology, School of Medicine, Aalborg University, Gistrup, Denmark; b Department of Gastroenterology & Hepatology, Mech-Sense, Clinical Institute, Aalborg University Hospital, Aalborg, Denmark; c Steno Diabetes Center North Denmark, Clinical Institute, Aalborg University Hospital, Aalborg, Denmark; d Department of Dermatology and Allergy, Herlev and Gentofte Hospital, Hellerup, Denmark; e Department of Clinical Medicine, University of Copenhagen, Copenhagen, Denmark

**Keywords:** Transcranial magnetic stimulation, Hypertonic saline-inducing pain, Histamine-inducing itch, Corticospinal excitability, Motor-evoked potentials, Pain catastrophizing scale, Itch catastrophizing scale

## Abstract

**Introduction:**

Pain and itch provoke different motor behaviors. Pain induces motor inhibition, whereas itch triggers an active scratching response. Acute pain is known to decrease corticospinal excitability, assessed by transcranial magnetic stimulation (TMS) through motor-evoked potentials (MEPs), but the effects of acute itch on bilateral corticospinal excitability are unknown.

**Objectives:**

We hypothesized that acute pain and itch differentially affect corticospinal excitability, decreasing and increasing MEPs, respectively.

**Methods:**

Nineteen healthy participants completed a 2-session, crossover study. Transcranial magnetic stimulation was applied targeting the right and left first dorsal interosseous (FDI) muscles. Motor-evoked potentials were recorded before, during experimentally induced pain (intramuscular hypertonic saline injection in the right FDI) or induced itch (cutaneous histamine application above the FDI), immediately after, and 30 minutes after provocation. Transcranial magnetic stimulation pulses were delivered every ∼7 seconds, and participants rated the pain and itch intensities using a numeric rating scale every ∼30 seconds.

**Results:**

Pain numeric rating scale scores were higher than itch scores from 0 to 120 seconds but lower at 390 seconds (all *P* < 0.05). Compared with baseline, both modalities reduced affected-hand MEPs (*P* = 0.003) with no changes in nonaffected-hand MEPs. Correlations between affected- and nonaffected-hand MEP reductions were found during pain (*P* = 0.009) and itch (*P* = 0.025).

**Conclusion:**

Opposite to the hypothesis, acute, experimental pain and itch induced similar corticospinal inhibition, suggesting a comparable effect on the motor output of the affected limb. This study found that the effects of acute pain and itch showed similar inhibition on bilateral corticospinal tract excitability.

## Abstract 2

The effects of acute pain and acute itch are similar on the bilateral corticospinal tract excitability, with corticospinal excitability decreasing in the targeted hand muscle.

## 1. Introduction

Pain and itch are generally considered to evoke different motor behaviors^33^ as pain can induce an inhibition of the motor system, whereas itch induces a scratching response.^13^ Despite these motor response differences, pain and itch show similar sensory manifestations, such as hyperalgesia (for pain) and hyperknesis (for itch), and nociceptive primary afferents may also convey itch.^10^ The sensory similarities have been frequently investigated,^42^ but the neural mechanisms driving the different motor behaviors have been explored less.

Motor responses during acute muscle pain have shown reduced maximal muscle contraction,^30^ faster development of exercise-induced fatigue,^34^ a redistribution of activity within muscles,^22^ and decreased movement variability.^4^ To investigate the neural substrates underlying motor changes, corticospinal excitability can be assessed using transcranial magnetic stimulation (TMS) of the primary motor cortex (M1),^17^ which has demonstrated an inhibitory effect on experimental pain.^26^ Transcranial magnetic stimulation can be delivered to M1 while the target area is subjected to experimental pain stimuli, such as hypertonic saline, capsaicin cream, or painful heat stimulation.^5^ These provocations have generally revealed a decrease in the peak-to-peak amplitudes of the motor-evoked potentials (MEPs) in the affected muscle or the muscle closest to the pain site during the painful stimulus,^21^ irrespective of the source of pain.^3^ This has been interpreted as an inhibitory mechanism, preventing further movements toward the insult.^9^ However, modulation patterns may vary depending on the muscle's functional role, with some studies reporting delayed facilitation in muscles engaged in withdrawal behaviors distant from the stimulus site.^37^ Given the contrasting motor behaviors, acute itch, which typically promotes active scratching, may be associated with facilitated rather than inhibited corticospinal excitability in the affected limb, in contrast to the suppressive effects observed during pain.

The effects of pain and itch are not limited to the affected limb but extend to the nonaffected limb.^14^ During pain or itch, compensatory motor behaviors appear, including movements in the nonaffected limb to protect the body or remove the source of pain or itch. For example, in functional contexts such as ankle sprains, individuals typically reduce weight-bearing on the painful side while increasing load and contact time on the opposite limb. This suggests a compensatory activation pattern that may also be reflected at the level of corticospinal excitability. Although no studies have directly investigated corticospinal excitability in the homologous muscle of the contralateral limb during tonic pain, 2 studies show a decrease in interhemispheric inhibition after acute muscle pain^1^ and sustained muscle pain.^31^ This decreased inhibition was seen from the M1 corresponding to the pain-affected muscle to the opposite M1 corresponding to the nonaffected limb.^31^ This reduced inhibition was observed from the motor cortex corresponding to the painful muscle toward the opposite hemisphere, suggesting possible facilitation of the nonaffected limb. Based on this, we hypothesized that corticospinal excitability in the nonaffected limb might increase as part of a compensatory motor strategy during both pain and itch.

The aim of this study was to investigate the effects of experimental acute muscle pain and superficial itch on bilateral corticospinal excitability assessed by TMS. As these modalities may evoke different motor responses, it is conceivable that they also produce different effects on the human corticospinal system. We hypothesized that when stimulated with a pruritogen, TMS-evoked MEPs in the affected first dorsal interosseous (FDI) muscle would increase to favor a scratch reflex. By contrast, when stimulating the same area with an algogen, TMS-evoked MEPs would decrease as a protective mechanism. In addition, a secondary hypothesis was made that, due to bilateral communication, the nonaffected hand would exhibit increased TMS-evoked MEPs during both induced sensations, reflecting a compensatory activation pattern. Furthermore, to explore whether changes in the affected hemisphere were mirrored in the contralateral hemisphere, we computed correlations between MEP amplitudes in the affected and nonaffected hands. This secondary analysis aimed to assess whether excitability changes were coordinated across hemispheres, which would support the notion of a bilateral motor strategy during aversive somatosensory experiences such as pain and itch.

## 2. Methods

### 2.1. Participants and study protocol

This study was conducted at the Center for Neuroplasticity and Pain, School of Medicine, Aalborg University, Aalborg, Denmark, in July–August 2024. The study was performed according to the Declaration of Helsinki, approved by the local ethics committee (Den Videnskabsetiske Komité for Region Nordjylland: N-20240004) and registered on clinicaltrial.gov (NCT06470737).

The sample size of this study was estimated based on a similar study observing the reduction in MEPs after injecting hypertonic saline into the FDI muscle compared with the extensor carpi radialis muscle (Site by Time interaction: F_3,36_ = 4.29, *P* = 0.01, $\eta_{p}^{2}$ = 0.26).^20^ The corresponding effect size for MEP reduction between the 2 conditions was calculated as 0.60. Using G*Power for statistical power analysis, with a desired power of 0.95, an alpha level of 0.05, and an effect size of 0.60, a minimum of 8 participants was necessary. However, as it was uncertain whether itch stimulation would increase or decrease MEPs, 21 volunteers were recruited for this study to ensure sufficient statistical robustness as well as account for possible dropouts.

Written consent forms were signed before the commencement of the experiment, and participants were excluded if they suffered from chronic and acute pain or itch or any neurological, musculoskeletal, or mental illnesses. Participants were also excluded if they took any analgesic medication as well as antihistamines of any kind. All participants were screened for contraindications to TMS^28^ and completed the following questionnaires: pain catastrophizing scale (PCS),^35^ Itch catastrophizing scale (ICS)^35^ (an adaptation of the PCS for itch), and positive and negative affects schedule (PANAS)^40^ to assess that no catastrophic thinking related to pain or itch was present during the experiment as well as no significant difference in the participants' positive and negative emotions between sessions.

The study used a randomized experimental crossover design and was conducted over 2 sessions of approximately 2 hours, separated by at least 2 days. On the first day of experiments, participants underwent 1 of 2 procedures chosen based on randomization: intramuscular injection of hypertonic saline or application of histamine. On the second day of experiments, participants underwent the same procedure, experiencing the other induction parameter. The order of the 2 stimuli was randomized on the first day using a digital randomization tool (https://wheelofnames.com/, last accessed August 26, 2024), which determined whether a participant would receive hypertonic saline or histamine first.

During the experiment, participants were seated comfortably in a chair with both hands resting on arm rests. For all data collection, especially during pain and itch inductions, participants were instructed to keep their hands still and as relaxed as possible. They were not allowed to scratch, rub, or shake the affected hand.

**Figure 1.**
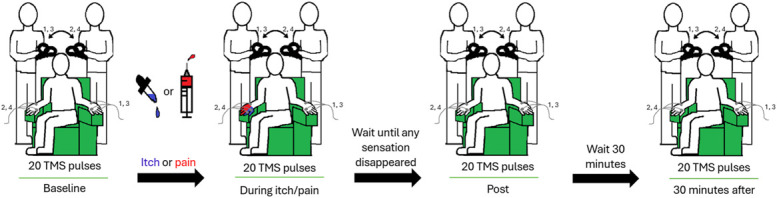
Experimental design. On the first day, participants underwent 1 of 2 procedures chosen based on randomization: intramuscular injection of hypertonic saline into the right first dorsal interosseus muscle or application of histamine on the dorsum of the right hand. On the second day of experiments, participants underwent the same procedure, experiencing the other induction condition. The TMS coil alternated between the right and left hemispheres with every pulse, and motor-evoked potentials were measured in the targeted muscle upon stimulus (numbers 1–4 illustrate the alternating pattern). TMS, transcranial magnetic stimulation.

At the start of each session, baseline measurements were made by delivering 20 TMS pulses at intervals of approximately 7 seconds (with 20% jitter), alternating between the left and right hemispheres between each stimulus, resulting in 10 pulses per hemisphere (Fig. 1). Whether the first pulse would be administered to the left or right hemisphere was chosen based on the same randomization tool as described for the order of stimulants. After the baseline, participants received either the hypertonic saline injection or histamine application, both stimulants to the right FDI muscle. Once participants reported a minimum pain or itch level of 3 on the numeric rating scale (NRS), 20 TMS pulses were administered as for the baseline. Participants rated their pain or itch using the NRS approximately every 30 seconds. Immediately after the pain and itch disappeared, and 30 minutes after, 20 TMS pulses were applied. For all conditions, the coil would alternate between the left and right hemispheres, resulting in each hemisphere being stimulated by every other pulse.

### 2.2. Hypertonic saline-induced pain

An intramuscular hypertonic saline injection of 0.1 mL sterile hypertonic saline (Natriumchlorid 7%, Aalborg University Hospital) into the right FDI muscle was performed to induce acute pain. The pain induced by this method is typically described as a cramping, burning, or tight muscular pain, and the saline can induce both localized and referred pain.^12^

### 2.3. Histamine-induced itch

Histamine dihydrochloride 1% (histamine dihydrochloride solution 10 mg/mL, ALK-Abellò A/S, Hørsholm, Denmark) was administered using standard allergy skin prick test lancets (Allergopharma, Hamburg, Germany)^15^ with a tip approximately reaching the dermo–epidermal junction.^11^ Two drops of histamine were placed on the skin above the right FDI muscle, one on either side of the electromyography (EMG) electrode. To decrease variability of the application method, a 120-g weight-calibrated custom-made device (Aalborg University, Aalborg, Denmark) was applied for 1 to 2 seconds. If this did not induce an itch level higher than 2, 2 additional drops would be applied.

### 2.4. Numeric rating scale

Numeric rating scale was used to assess the intensity and duration of itch and pain. The NRS is a verbal assessment of either pain or itch sensations. Immediately after the induction of pain or itch, the subjects rated the perceived sensation on a scale from 0 to 10, with 0 representing no sensation and 10 representing the worst imaginable sensation.^16^ The given ratings were documented when the sensation reached a minimum of 3, after which the TMS pulses would begin to be administered. Subsequently, the participants assessed the sensation after every third TMS pulse.

### 2.5. Motor evoked potentials

Single-pulse TMS was delivered using a monophasic TMS stimulator (Magstim M200^2^, Magstim Co. Ltd, Dyfed, United Kingdom) with a figure-of-eight–shaped coil (D70^2^ coil, Magstim Co. Ltd) to elicit TMS-induced MEPs in the left and right FDI muscles recorded with surface EMG. Surface disposable silver/silver chloride adhesive electrodes (Ambu Neuroline 720, Ballerup, Denmark) were placed on both hands on the surface of the skin directly above the FDI muscle parallel with the underlying muscle fibers as well as on the dorsal surface of the proximal phalanx of the index fingers. Reference electrodes were placed on the styloid processes of the ulnas. The EMG signals were band-pass filtered at 5 Hz to 1 kHz, sampled at 2 kHz, and digitized by a 16-bit data-acquisition card (NI6122X; National Instruments, West Haven, CT).

To locate the target areas, the left and right M1 in each participant was identified by stimulating both the left and right hemispheres near the central sulcus with TMS pulses until MEP responses in the FDI muscles were seen. The “hot spots” were determined based on the largest MEPs recorded through EMG. To ensure precise targeting of the hot spots during stimulation, a 3D reconstruction of the brain was generated using the Brainsight software (Brainsight TMS Neuronavigation, Rogue Research Inc., Montréal, Canada) using the navigated brain stimulation system, which was scaled to fit the subject's head. An optical tracking system and the navigated brain stimulation system were used to continuously calibrate the subject's head position. The TMS coil was equipped with a coil tracker, allowing the neuronavigation system to monitor and register changes in angle and position with an accuracy of 0.1 millimeters, ensuring consistent targeting between sessions.^29^

Resting motor thresholds (rMT) for each hotspot were measured by finding the lowest intensity to evoke a MEP ≥ 50 μV. The motor threshold assessment software tool MTAT 2.0 was used, which applies an adaptive threshold-hunting algorithm. As a result, the number of stimuli varied slightly across participants depending on the convergence speed of the algorithm.^18^

After determining the rMT, the stimulation intensity was set to 120% of the individual rMT for the left hemisphere to ensure consistent suprathreshold stimulation while accounting for individual variability in corticospinal excitability. This choice was also based on previous studies using hypertonic saline, where 120% of the rMT was commonly used and shown to reliably reveal corticospinal inhibition.^20,32^

### 2.6. Statistics

Statistical analysis was performed on SPSS software (v26, IBM Corporation, Armonk, NY). Data are shown as mean ± standard error of the mean (SEM) unless otherwise stated, and a *P*-value < 0.05 was considered statistically significant. The Shapiro–Wilk normality test was performed, and QQ plots were examined to check the normality. The NRS measurements generally followed a normal distribution, but because most peak-to-peak MEP measurements were not normally distributed, log transformation was applied to normalize the data.

Numeric rating scale variables were analyzed using repeated-measures analysis of variance (RMANOVA), with Condition (Pain, Itch) and Time (immediately after, 30, 60, 90, 120, 150, 180, 210, 240, 270, 300, 330, 360, 390 seconds) as within-subject factors, including their interaction (Condition × Time). Motor-evoked potential variables were similarly analyzed using RMANOVA, with Condition (Pain, Itch) and Time (baseline, during pain or itch, immediately postsensation, and 30 minutes postsensation) as within-subject factors. Interaction effects between Condition and Time were also included in the models, and effect sizes were reported using partial eta squared ($\eta_{p}^{2}$). The Greenhouse−Geisser approach was used to correct against violations of sphericity. Where appropriate, post hoc pairwise analyses were performed with Bonferroni-corrected multiple comparisons.

Spearman correlation analyses were performed on the percentage ratio of peak-to-peak amplitudes, calculated as (pain or itch)/baseline × 100, to examine the relationship between MEP changes in the affected and nonaffected hands during itch and pain. This analysis was conducted to explore whether excitability changes were coordinated across hemispheres, consistent with the hypothesis of bilateral motor modulation. The percentage ratio was used for the correlation analysis because it provides a standardized and directionally consistent metric of change relative to baseline, minimizing the distortions that can arise from negative values or skewed distributions in percent change calculations.^39^

Finally, a paired *t* test was conducted on the PANAS for both sessions to check for the possible confounding of the participants' different positive and negative emotions during the 2 sessions.

## 3. Results

### 3.1. Positive and negative affects schedule and numeric rating scale scores

One of the 21 participants withdrew from the experiment before the experimental session with hypertonic saline injection and another participant was excluded because they did not feel any pain 3 minutes after the injection, resulting in 19 participants (9 women) completing both sessions successfully. The age, height, and weight were, respectively: 25 ± 5 years old, 177 ± 10 cm, and 70 ± 13 kg. The paired *t* test on PANAS scores found no significant changes between the 2 sessions (*P* = 0.068).

**Figure 2.**
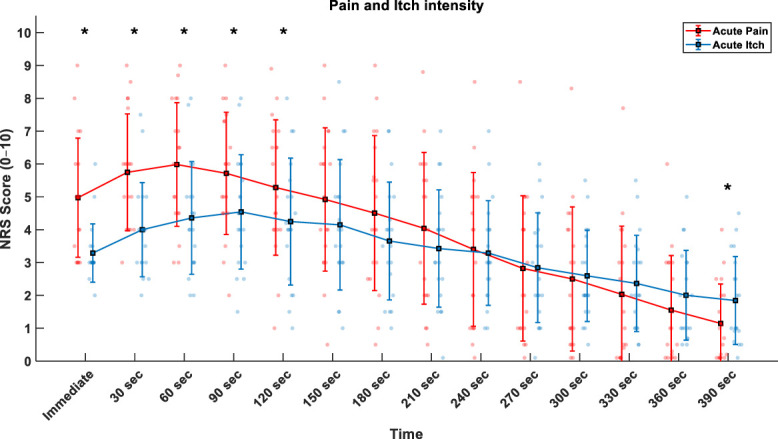
Mean ± SD of pain and itch numeric rating scale (NRS) over time for injection of hypertonic saline into the right first dorsal interosseus muscle and histamine on the dorsum of the right hand (*P < 0.05 Bonferroni-corrected).

For NRS scores, the 2-way RMANOVA revealed a Condition × Time interaction (F_3.0, 56.5_ = 9.774; *P* < 0.001; $\eta_{p}^{2}$ = 0.352) and a Time effect (F_3.0, 56.1_ = 64.751; *P* < 0.001; $\eta_{p}^{2}$ = 0.783). Post hoc pairwise analyses found that NRS scores were significantly higher for pain compared with itch immediately after the application of the sensory conditions (Δ 1.7 ± 0.4, *P* < 0.001), at 30 seconds (Δ 1.7 ± 0.4, *P* < 0.001), 60 seconds (Δ 1 ± 0.5, *P* = 0.005), 90 seconds (Δ 1.2 ± 0.4, *P* = 0.015), and 120 seconds (Δ 1.0 ± 0.5, *P* = 0.034) (Fig. 2). By contrast, after 390 seconds, NRS scores for itch were significantly higher than for pain (Δ 0.7 ± 0.3, *P* = 0.049). For pain, the NRS scores peaked after 60 seconds (5.9 ± 0.4), while itch peaked after 90 seconds (4.5 ± 0.4).

### 3.2. Motor-evoked potentials—affected and nonaffected corticospinal excitability

**Figure 3.**
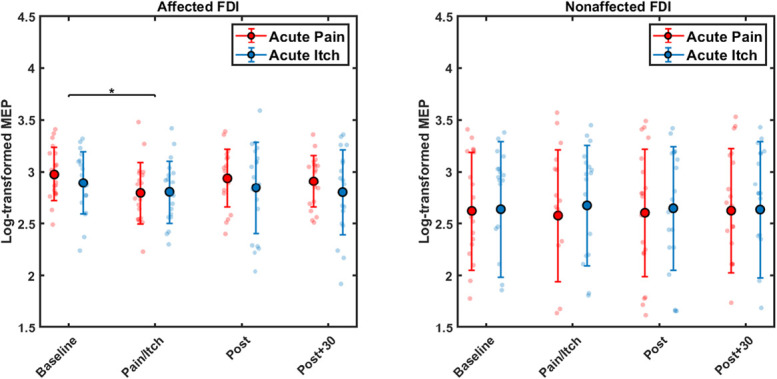
Mean ± SD of log-transformed motor-evoked potentials (MEPs) in the affected and nonaffected first dorsal interosseous (FDI) muscles after hypertonic saline (Acute Pain) and histamine (Acute Itch) stimulation. Asterisks indicate a significant main effect of time across conditions (*P* < 0.05).

For the MEPs, the 2-way RMANOVA in the affected FDI muscle revealed a time effect (F_3,54_ = 5.696, *P* = 0.002, $\eta_{p}^{2}$ = 0.240) and no Condition × Time interaction was found (F_3,54_ = 1.100, *P* = 0.357, $\eta_{p}^{2}$ = 0.058). Post hoc pairwise analyses revealed a decrease from baseline to pain/itch (*P* = 0.003) (Fig. 3). Specifically, the decrease was −328.6 μV ± 137.9 (23% ± 10) for pain and −147.4 μV ± 65.6 (10% ± 11) for itch. No significant differences were observed between any other time points, including baseline to immediately post (*P* = 1.000), baseline to 30 minutes post (*P* = 0.271), pain/itch to immediately post (*P* = 0.191), pain/itch to 30 minutes post (*P* = 0.824), or post to 30 minutes post (*P* = 1.000).

No Condition × Time interaction or Time effect was found by the 2-way RMANOVA for the MEP changes in the nonaffected FDI muscle (Condition × Time interaction F_3,54_ = 0.592, *P* = 0.623, $\eta_{p}^{2}$ = 0.032; Time effect F_3, 54_ = 0.007, *P* = 0.999, $\eta_{p}^{2}$ = 0.000) (Fig. 3). The non–log-transformed data are summarized in Table 1.

**Table 1:** Mean (±SEM, N = 19) parameters related to motor-evoked potentials of the first dorsal interosseous muscles (μV).

	Condition	Baseline	During	Post	30 min Post
MEPs affected FDI	Pain	1120 ± 147	792 ± 145	1041 ± 134	941 ± 113
	Itch	954 ± 123	807 ± 134	1056 ± 200	900 ± 152
MEPs nonaffected FDI	Pain	798 ± 174	826 ± 222	879 ± 220	881 ± 223
	Itch	831 ± 151	875 ± 176	861 ± 179	872 ± 175

FDI, first dorsal interosseous; MEP, motor-evoked potential.

### 3.3. Correlations

**Figure 4.**
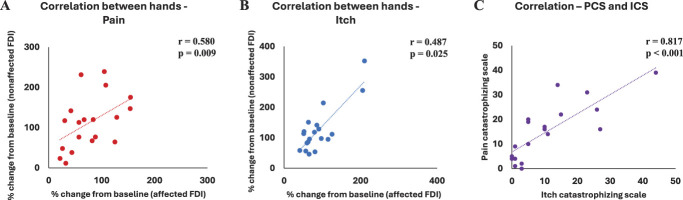
(A) Correlation of percentage changes in motor-evoked potentials (MEPs) between the affected and nonaffected first dorsal interosseous (FDI) muscles during pain (percentage change from baseline); (B) correlation of percentage changes in MEPs between the affected and nonaffected FDI muscles during itch (percentage change from baseline); (C) correlation between itch catastrophizing scale and pain catastrophizing scale.

Spearman correlation analysis of the percentage changes in peak-to-peak amplitudes from baseline revealed a correlation during pain (r = 0.580, *P* = 0.009; Fig. 4A) and a correlation between MEP changes in the affected and nonaffected hands during itch (r = 0.487, *P* = 0.025; Fig. 4B). No significant correlations between MEP changes in the affected and nonaffected hands were observed at any other time points, including post (itch, *P* = 0.112; pain, *P* = 0.135) or 30 minutes post (itch, *P* = 0.352; pain, *P* = 0.435). A significant correlation was observed between PCS scores and ICS scores (r = 0.817, *P* < 0.001—Fig. 4C).

## 4. Discussion

This study showed that experimental acute muscle pain and experimental acute histamine-induced itch inhibited corticospinal excitability in a similar way. Both sensory manifestations led to a reduction in MEPs, with MEP changes in the affected FDI muscle positively correlating with those in the nonaffected FDI muscle. Taken together, the present findings suggest that the effects of acute pain and itch were similar on the bilateral corticospinal tract excitability.

### 4.1. Acute pain and acute itch models

In this study, 2 common experimental provocation approaches were selected to induce acute pain and acute itch, as previous research has shown that these 2 models produce comparable subjective sensory temporal profiles, characterized by an immediate onset after the application, a rapid peak, a similar duration, and the absence of postsensory effects after around 10 minutes.^2,20^ However, the present results revealed that the 2 experimental models exhibited slightly distinct temporal profiles: hypertonic saline induced a faster increase in the sensation, peaking within 60 seconds, followed by a faster decline with almost no pain after 6 to 7 minutes. By contrast, the histamine-induced itch showed a slower peak, after around 90 seconds, a slower decrease, and a prolonged sensation lasting more than 6 to 7 minutes. Even so, the other acute pain models commonly used to study corticospinal excitability inhibition, such as capsaicin cream,^41^ heat pain,^6^ or laser-induced pain,^38^ differ significantly further in temporal profile which makes comparing them with acute itch more challenging. As an alternative itch model, cowhage-induced itch was a potential option for this study as this method activates nonhistaminergic pathways in contrast to histamine activating the histaminergic pathways. However, cowhage may induce both itch and a slight pain perception,^2^ which could confound the results. As a result, the 2 most compatible models for comparing acute pain and acute itch were considered hypertonic saline injection-induced pain and histamine-induced itch, but future studies could compare the effects of histaminergic and nonhistaminergic itch on corticospinal excitability.^24^

The study showed a significant correlation between the ICS and PCS scores—the participants' catastrophizing thoughts associated with pain and itch, confirming that both pain and itch are largely affected by emotions and mental state.^23,25^

### 4.2. Bilateral corticospinal excitability during acute pain and acute itch

The study showed a significant decrease in corticomotor excitability of the affected FDI muscle in response to both hypertonic saline-induced acute pain and histamine-induced acute itch, contrasting the main hypothesis. The intramuscular injection of hypertonic saline has been widely used in previous studies, showing an inhibition of corticospinal excitability,^26^ although some studies found no reduction.^7,27^ The assessed MEP percentage reduction in corticospinal excitability matches findings from previous studies.^21^ The mechanisms underlying corticospinal excitability inhibition are still debated. Peripheral M-wave amplitudes, reflecting sarcolemma excitability and intramuscular action potential, remain unchanged during experimental acute pain,^8^ suggesting that the origin of the inhibition is at the spinal or cortical level. Studies investigating spinal excitability have reported a depression of the excitability of the motoneurons^21,36^ and changes in inhibitory and facilitatory intracortical circuits in response to acute pain, as assessed by paired-pulse TMS paradigms of hand musculature.^32^ This suggests both cortical and spinal roles in the MEP inhibition. While the inhibitory effects of acute pain on MEPs have also been found by several other studies,^19,20,36^ this is the first study to investigate the effect of acute itch on the corticomotor excitability. Contrary to our hypothesis, a reduction in corticospinal excitability was observed by itch provocations. This finding suggests that the mechanisms of itch may be more closely related to those of pain, but further studies are necessary, including assessments of peripheral M-wave, spinal reflexes, intracortical inhibition, and/or facilitation.

The results of the secondary hypothesis, stating that the nonaffected hand would exhibit increased TMS-evoked MEPs during the induced sensations, reflecting motor responses such as compensatory mechanisms and scratching associated with pain and itch, were ambiguous. No significant changes in MEP amplitudes were observed in the nonaffected FDI muscle at any time point. However, significant correlations between changes in MEP amplitudes in the affected and nonaffected hands during both pain and itch may suggest the presence of interhemispheric communication during both modalities.^1^ This could indicate that itch often engages a contralateral response, typically involving the opposite limb to scratch the affected area. Similarly, during pain, the contralateral limb may be recruited to compensate for reduced motor output in the affected limb. However, caution is advised in interpreting these findings because several factors can have affected these results. For one, the participants were instructed to neither scratch nor rub their hands during the itch and pain stimuli. This could partly be responsible for inhibiting a muscle response in the nonaffected hand as they would likely focus some of their attention on keeping this still despite the desire to scratch or rub. Moreover, the modulation of cortical motor output can vary depending on the function of the muscle and its proximity to the stimulus.^37^ Future studies should include assessments of muscles located further from the stimulation site to determine whether similar patterns are observed across functionally distinct and spatially distant muscle groups.

## 5. Limitation

This study has several limitations: First, acute pain and acute itch have slightly different temporal profiles, which is a challenging problem when comparing experimental pain and itch. Based on the literature, the 2 temporal profiles were the most comparable. However, because both stimulus modalities induced an inhibition in the corticospinal excitability, these differences in intensity and duration may not be highly relevant.

A second limitation is the absence of additional neurophysiological assessments beyond MEP amplitude, such as H-reflexes, intracortical inhibition and facilitation (eg, short-interval intracortical inhibition, intracortical facilitation, and the cortical silent period), or interhemispheric inhibition measures. Including these parameters would have provided a more comprehensive understanding of the cortical and spinal mechanisms modulating motor output during pain and itch. However, as this study is the first to investigate the effects of acute itch on corticospinal excitability, we chose to focus on MEPs as a well-established index of corticospinal output. The current findings highlight the need for future studies to incorporate broader TMS protocols to better elucidate the neural mechanisms underlying motor control in response to acute pain and itch.

Finally, a limitation of this study is the absence of self-report measures assessing the urge to move, such as the urge to scratch or withdraw. Including such measures in future work could help link subjective sensorimotor drive more directly to the observed modulation of corticospinal excitability.

## 6. Conclusion

Acute itch, as well as acute pain, inhibited the TMS-induced MEPs in the affected FDI muscle. In addition, a significant correlation was found between the affected and nonaffected FDI muscles during both acute pain and acute itch. The interhemispheric communication is congruent with itch being a contralateral reflex, which uses the opposite limb to scratch the affected area. Furthermore, a significant correlation was found between the catastrophizing thoughts of pain and itch, indicating that a person's threshold for acute itch may be linked to their threshold for acute pain.

## Disclosures

The authors have no conflict of interest to declare.
